# Automated Fibrosis-4 Index: Simplifying Non-Alcoholic Fatty Liver Disease for Diabetologists

**DOI:** 10.3390/medicina60081278

**Published:** 2024-08-08

**Authors:** Mona H. Ismail, Reem Al Argan, Yasir Elamin, Murtaga Makki, Lameya Alsheekh, Jaber Alelyani, Zahra Hadhiah, Zahrah Aljidhr, Nazih Alkhatam, Hind Alfaddagh, Alanoud Alanazi, Shaya Alqahtani

**Affiliations:** 1 Division of Gastroenterology, Department of Internal Medicine, King Fahd Hospital of the University, Al-Khobar 31952, Saudi Arabia; murtagaja@gmail.com (M.M.); alsheekh.lameyaa89@hotmail.com (L.A.); jaberalelyani89@gmail.com (J.A.); zahrahhj@gmail.com (Z.A.); halfaddagh@moh.gov.sa (H.A.); 2 College of Medicine, Imam Abdulrahman Bin Faisal University, Dammam 31441, Saudi Arabia; rjalarqan@iau.edu.sa (R.A.A.); yaelamin@iau.edu.sa (Y.E.); ahalanazi@iau.edu.sa (A.A.); saalqahthani@iau.edu.sa (S.A.); 3 Division of Endocrine, Department of Internal Medicine, King Fahd Hospital of the University, Al-Khobar 31952, Saudi Arabia; zhadhiah@moh.gov.sa (Z.H.); nalkhatam@moh.gov.sa (N.A.); 4 Department of Internal Medicine, King Fahd Hospital of the University, Al-Khobar 31952, Saudi Arabia

**Keywords:** NAFLD, advanced fibrosis, type 2 diabetes, electronic medical records, fibrosis-4, Saudi Arabia

## Abstract

*Background and Objectives*: Patients with type 2 diabetes (T2D) have a high prevalence of non-alcoholic fatty liver disease (NAFLD) (55%) and are at increased risk for developing non-alcoholic steatohepatitis, a severe form of NAFLD. Early detection of advanced fibrosis in patients with T2D and NAFLD is crucial and can prevent progression to chronic liver disease, cirrhosis, and hepatocellular carcinoma. However, screening for liver disease and risk-stratification pathways are not established in patients with T2D. We evaluated the efficacy of using the automated fibrosis-4 (FIB-4) index in routine clinical settings to identify patients requiring further specialist evaluation. *Materials and Methods*: In this prospective cohort study, individuals diagnosed with T2D were recruited from diabetes clinics at a tertiary university hospital. Demographic, clinical, and laboratory data were comprehensively collected. The FIB-4 index was automatically calculated and integrated into the hospital’s electronic medical records (EMRs), which were then stratified by age. Patients with advanced fibrosis (FIB-4 index ≥ 1.3) were referred to a specialist. Student’s *t*-test or the Mann–Whitney U test was used to analyze variables associated with advanced fibrosis. Logistic regression was used to identify predictors of advanced fibrosis. *Results*: Among the 318 patients with T2D, 9.7% had advanced fibrosis. The majority were females (54.7%) and Saudi nationals (89.6%). Several factors, including age, platelet count, total bilirubin, serum albumin, total cholesterol, low-density lipoprotein, transaminases, and gamma-glutamyl transferase (GGT), showed significant associations with advanced fibrosis (all *p* < 0.05). Older age, elevated total bilirubin and GGT levels, and prolonged international normalized ratio emerged as independent predictors of advanced fibrosis. *Conclusions*: Integrating the FIB-4 index into the EMR during the routine care of patients with T2D proved to be a valuable tool in effectively identifying individuals at risk of advanced fibrosis. Our findings emphasize the need for further research to refine screening strategies in this high-risk population.

## 1. Introduction

Non-alcoholic fatty liver disease (NAFLD) has emerged as a significant global health concern, representing the leading cause of chronic liver disease [1]. Its prevalence is estimated at 24% worldwide [2], but it doubles among individuals with type 2 diabetes (T2D), reaching 55.5% [3]. T2D significantly increases the risk of a more severe form of NAFLD known as non-alcoholic steatohepatitis (NASH), which can lead to advanced fibrosis (bridging fibrosis and cirrhosis). Patients diagnosed with NASH are at risk of progressing to cirrhosis and primary liver cancer [4]. Clinicians must recognize that individuals with T2D are particularly susceptible to developing NASH. Additionally, patients with T2D and NAFLD often remain asymptomatic and may have normal liver enzyme levels, making liver biopsy necessary for diagnosis. This disease often goes undetected and is commonly underdiagnosed by healthcare providers [5,6]. Recognizing the importance of early detection, international clinical guidelines such as those established by the European Association for the Study of the Liver (EASL) [7], the American Association for the Study of Liver Diseases (AASLD) [8], and the American Diabetes Association [9] advocate regular screening for NAFLD in patients with T2D who exhibit fatty liver on ultrasonography or elevated liver enzymes.

Determining the stage of liver fibrosis is crucial for predicting future progression to cirrhosis, liver-related mortality, and hepatoma [10,11]. Currently, liver biopsy serves as the gold standard to diagnose liver fibrosis; however, its invasive nature, association with pain and discomfort, dislike by patients, and sampling variability pose significant constraints [12,13]. Given the anticipated rise in NAFLD prevalence and healthcare burden due to the ongoing epidemic of diabetes and obesity, there is an urgent need for a well-validated, simple, and easy non-invasive test (NIT) to help identify advanced fibrosis in patients with T2D during routine clinical care. The fibrosis-4 (FIB-4) index is a well-validated NIT that can help identify advanced fibrosis in diabetic patients with NAFLD in the clinic [14,15,16] and is recommended by international guidelines [7,8,9]. The FIB-4 has high negative predictive values for ruling out advanced liver fibrosis due to NAFLD and other chronic liver diseases. Patients with abnormal FIB-4 scores can subsequently undergo more specific non-invasive fibrosis tests, such as FibroScan, which can be performed in specialized facilities depending on the accessibility of such tests. However, studies investigating the development of clinical care pathways incorporating such NITs within the electronic medical record (EMR) of patients at risk for advanced fibrosis in diabetes clinics remain scarce. This study aimed to evaluate the effectiveness of integrating automated FIB-4 index calculation in diagnosing advanced fibrosis in patients with T2D and NAFLD, with the goal of facilitating early referral to hepatologists or specialists for further evaluation.

## 2. Materials and Methods

### 2.1. Study Design

This prospective cohort study included all adult patients ≥18 years with T2D who signed informed consent during the outpatient visits at the diabetes clinic at King Fahad Hospital of the University between August 2021 and February 2023. We excluded patients with viral hepatitis, alcohol consumption, use of steatogenic medication (such as amiodarone, tamoxifen, methotrexate, or steroids), pregnancy, other hepatic diseases, hepatoma, or decompensated cirrhosis. 

### 2.2. Data Extraction

All participants underwent standard anthropometric measurements, serum biochemical tests, and a review of their medical histories. Blood samples were collected after a fasting period of at least 12 h. We collected demographic, clinical, and laboratory data about age, sex, nationality, body mass index (BMI), hemoglobin level, platelet count, liver enzymes (alanine transaminase, ALT; aspartate transaminase, AST; gamma-glutamyl transferase, GGT), albumin, international normalized ratio (INR) of prothrombin time, total cholesterol (CHOL), low-density lipoprotein (LDL), high-density lipoprotein (HDL), triglycerides (TG), hemoglobin A1c, the presence of hypertension, and medications used. The hepatic steatosis index (HSI) was also calculated at the diabetes clinic. The HSI is a non-invasive mathematical model designed to predict hepatic steatosis. HSI = 8 × ALT/AST + BMI + 2 (if T2D) + 2 (if female). An HSI score above 36 indicates the presence of hepatic steatosis, while a score below 30 indicates its absence [17]. Patients with steatosis by HSI were referred for further evaluation by abdominal ultrasound.

### 2.3. FIB-4 Index Risk Stratification

**Figure 1:**
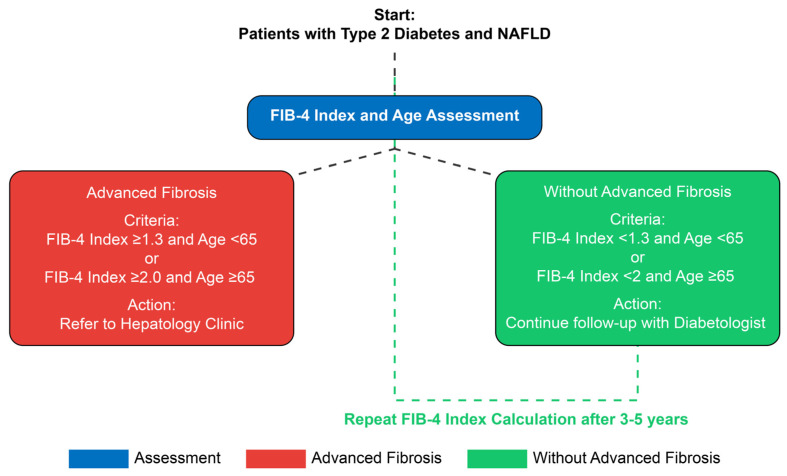
The clinical care pathway for patients with type 2 diabetes and NAFLD.

Before initiating the study, health information system specialists integrated the FIB-4 index algorithm into the EMR system (QuadraMed^®^, Plano, TX, USA). The FIB-4 index was automatically computed for all patients attending the diabetes clinic according to the following formula and adjusted for age: (Age [yr] × AST [U/L])/((PLT [10^9^/L]) × (ALT^1/2^ [U/L])) [18,19]. Patients with T2D and NAFLD were divided into two groups: advanced fibrosis and without advanced fibrosis. Individuals with a FIB-4 index ≥ 1.3 and age < 65 years or FIB-4 index ≥ 2.0 and age ≥ 65 years were identified as having advanced fibrosis (indeterminate- and high-risk scores) and were referred by the diabetologist to the hepatology clinic (Figure 1) for further workup. Patients with a FIB-4 index of <1.3 and age < 65 years or FIB-4 index < 2 and age > 65 years were at low risk for advanced fibrosis; they continued to follow up with their diabetologist and underwent a repeat FIB-4 index calculation after 3–5 years. Further stratification was based on ALT levels (normal: ≤40 IU/L and elevated: >40 IU/L).

### 2.4. Statistical Analysis

Descriptive statistics were employed, with continuous variables presented as the mean [standard deviation] (SD) or median and interquartile range (IQR) and categorical variables expressed as numbers or percentages. The Shapiro–Wilk test was used to assess the normality of the data. The chi-square or Fisher’s exact test was used for categorical variables, and the Student’s *t*-test or Mann–Whitney U test was used for continuous data. All analyses compared the groups with advanced fibrosis and those without advanced fibrosis. Correlations between the FIB-4 index and various clinical characteristics were examined. Multivariate logistic regression with backward selection identified independent predictors of advanced fibrosis using the FIB-4 index, with variables removed if their *p*-value exceeded 0.05. Associations were expressed as odds ratios (ORs) with 95% confidence intervals (CIs). The following variables were entered into the base model: age, sex, BMI categories, hypertension, ALT, AST, GGT, hemoglobin, platelet count, total cholesterol, HDL, LDL, bilirubin, albumin, and INR. A *p*-value < 0.05 was considered statistically significant, and all analyses were performed using the statistical software SPSS (version 25.0, IBM Corp., Armonk, NY, USA).

## 3. Results

### 3.1. Baseline Characteristics

Among the 318 patients meeting the eligibility criteria, the mean age was 54.8 (±13.3) years, with 54.7% being female and most participants being Saudi nationals (89.6%). Overall, 263 (84.0%) of our patients had an HSI > 36, indicating the presence of steatosis (Table 1).

### 3.2. Factors Associated with Advanced Fibrosis According to the FIB-4 Index

**Figure 2:**
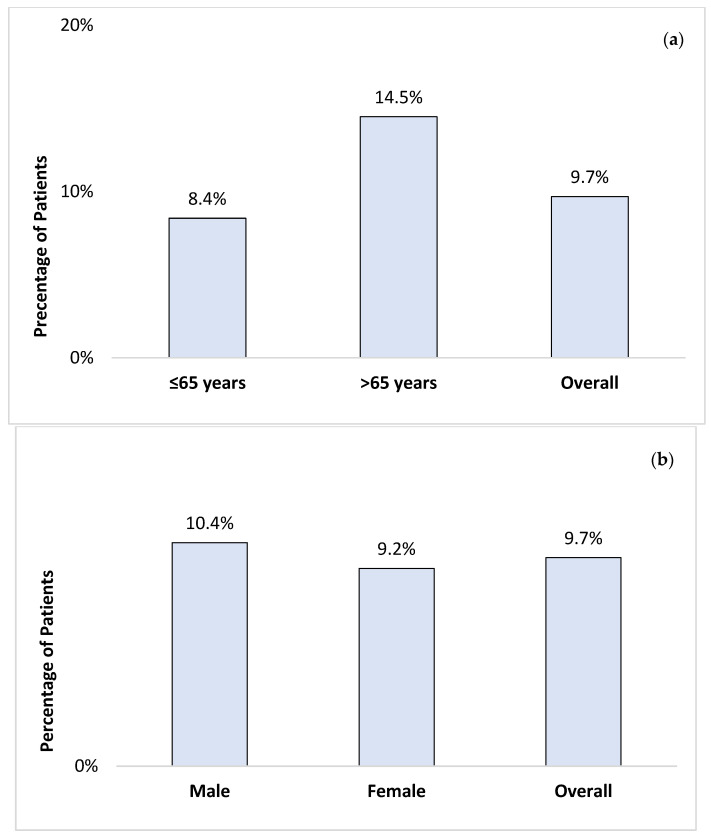
The prevalence of advanced fibrosis, using the FIB-4 index, by age (**a**) and gender (**b**) among patients with T2D attending the diabetes clinics.

Among the total cohort, 9.7% of patients had advanced fibrosis. Of these, 14.5% were over 65 years old, and 10.4% were male (Figure 2). Patients with T2D and advanced fibrosis were notably older, with a mean age of 62.5 (±10.9) years (*p* < 0.05) and a mean BMI of 30.1 (±4.9) kg/m^2^. In contrast, patients without advanced fibrosis had a mean age of 53.9 (±13.3) years and a mean BMI of 32.20 (±6.8) kg/m^2^. Based on the FIB-4 index, advanced fibrosis was significantly associated with age, platelet count, total bilirubin, serum albumin, AST, ALT, GGT, total cholesterol, LDL, and INR (all *p* < 0.05) (Table 1). Platelet count was lower in the advanced fibrosis group at 167 (133–194) × 10^9^/L (*p* < 0.001).

### 3.3. Advanced Fibrosis According to the Liver Enzymes

**Figure 3:**
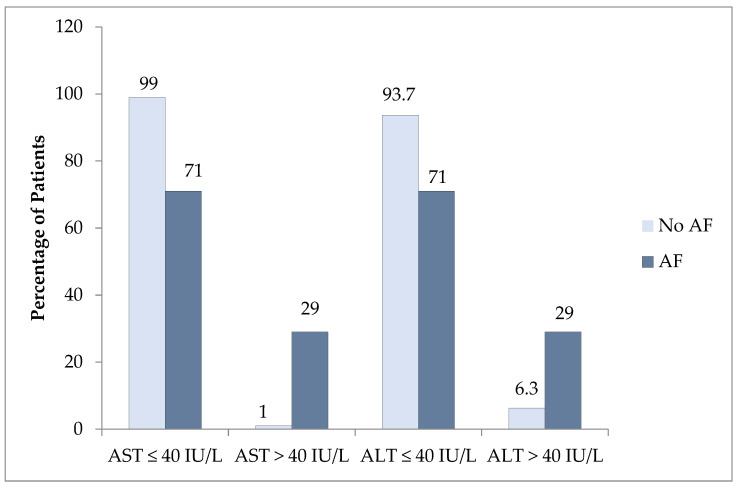
The proportion of patients with elevated AST or ALT with and without advanced fibrosis (AF) as assessed by the FIB-4 index.

Liver transaminases, ALT, and AST were normal in patients with advanced fibrosis, with a median ALT of 26 (IQR: 16–53 IU/L; *p* < 0.005) and AST of 25 (IQR: 20–45 IU/L; *p* < 0.001) (Table 1). We performed subgroup analysis based on the ALT levels (>40 IU/L or ≤40 IU/L) and found that 71% of patients with advanced fibrosis had normal ALT levels (Figure 3). In contrast, 6.3% and 1% of patients without advanced fibrosis had elevated ALT and AST levels, respectively.

### 3.4. Predictors of Advanced Fibrosis in Patients with T2D and NAFLD

Multivariate regression analysis included all variables with a trend toward association for advanced liver fibrosis in the univariate analysis. Advanced liver fibrosis showed significant associations with older age (in years), elevated total bilirubin (in mg/dL), elevated GGT (in IU/L), and prolonged INR. For every one-year increase in age, the odds of advanced liver fibrosis increased by 6% after adjusting for other variables. Patients with elevated bilirubin levels were 4.86 times more likely to develop advanced liver fibrosis than those with normal bilirubin levels (OR: 4.86; 95% CI, 1.55–15.19; *p* = 0.007). Similarly, elevated GGT (OR: 5.16; 95% CI, 2.10–12.68; *p* < 0.001) and prolonged INR (OR: 4.18; 95% CI, 1.17–14.96; *p* = 0.028) were predictive of advanced fibrosis (Table 2).

## 4. Discussion

This prospective study aimed to evaluate a clinical care pathway for identifying advanced fibrosis in patients with T2D. By integrating the automated calculation of the FIB-4 index into the EMR of patients, advanced fibrosis was identified in 9.7% of patients, including those with normal liver transaminase levels. The FIB-4 index revealed that advanced fibrosis was significantly associated with age, platelet count, total bilirubin, serum albumin, liver enzymes, lipid profile, and INR, while logistic regression analyses identified age, elevated bilirubin, elevated GGT, and prolonged INR as predictors of advanced fibrosis. This integration could reduce unnecessary referrals, help diabetologists efficiently identify patients with advanced fibrosis, and guide referrals to hepatologists or specialists for further evaluation. To our knowledge, this is the first reported clinical care pathway for NAFLD in T2D patients using automated FIB-4 calculations in a clinical setting.

Patients with T2D are more likely to develop NASH and can present with normal liver enzymes. Gawrieh and colleagues examined 534 patients diagnosed with NAFLD who had normal ALT levels and found that 19% had NASH with clinically significant or advanced fibrosis (F2-F3), and 7% had cirrhosis, with T2D emerging as the strongest predictor of NASH [5]. Similarly, another study demonstrated that obese patients with T2D had a higher risk of NASH despite normal liver enzyme levels [15]. Additionally, a study reported that only 15% of patients with T2D and NAFLD showed moderate to advanced liver fibrosis (≥F2), with elevated liver enzymes present in a minority of patients. [16]. In line with previous research, our study found that 9.7% of patients with T2D had advanced fibrosis, with 71% of these patients having normal ALT levels. Conversely, 29% of patients without advanced fibrosis had elevated ALT. This discrepancy underscores the limitations of relying solely on liver enzymes to diagnose liver fibrosis, highlighting the need for more vigilant screening strategies in managing patients with T2D and NAFLD.

Advanced fibrosis, assessed by the FIB-4 index, significantly correlates with decreased platelet counts and elevated levels of total bilirubin, AST, and GGT. Similar findings were reported in a study where high levels of AST and GGT, along with low platelet counts, predicted advanced fibrosis or cirrhosis in patients with NAFLD [20]. Given that the FIB-4 index incorporates age and platelet count, our results reinforce the importance of these parameters in the assessment of advanced fibrosis. Moreover, the diagnosis of liver disease is often delayed in patients with T2D, complicating the early detection and management of advanced fibrosis. This highlights the need to incorporate liver health assessments into diabetes management protocols to improve risk assessment and the timely diagnosis of liver complications [21]. 

The prevalence of NAFLD increases with age [22,23], and our study showed it was more common in older women than in men (54.7% vs. 45.3%). A previous study also reported a higher incidence of NAFLD in older women, with 24% having advanced fibrosis at diagnosis, although only 25% of the cohort had T2D [24]. This gender disparity likely arises from factors such as sex hormones, changes in body fat distribution in postmenopausal women, alcohol consumption effects in men, and physiological differences. 

Clinical guidelines recommend screening patients with T2D for NAFLD, yet its implementation remains challenging. In a study involving 467 patients, primary care providers screened T2D patients for NAFLD by integrating FIB-4 calculation into EMRs. Patients with FIB-4 >1.3 and age ≤65 years or FIB-4 >2.0 and age <65 years underwent additional reviews using FibroScan. Approximately 18.5% showed elevated FIB-4; a specialist review identified an additional 4.5% with NAFLD [25]. Routine laboratory results, age, and the FIB-4 index hold promise in identifying advanced fibrosis in T2D patients with NAFLD, potentially reducing unnecessary referrals and aiding in patient identification and referral for further care [15,16,25].

Our study has notable strengths, including the recruitment of a unique NAFLD population who have never consumed alcohol, eliminating a common confounding factor in NAFLD research. Additionally, our prospective design and use of the FIB-4 index enhance the risk stratification of advanced fibrosis in NAFLD patients in diabetes clinics, augmenting this study’s value. However, our study has some limitations, notably the absence of confirmatory second testing to validate advanced fibrosis identified by the FIB-4 index. Our primary objective was to evaluate the feasibility and effectiveness of incorporating the FIB-4 index into a clinical care pathway for patients with T2D. This integration aims to streamline referrals and minimize unnecessary procedures by accurately identifying at-risk patients. Although the FIB-4 index is useful for initial screening, it does not provide a definitive diagnosis of advanced fibrosis, warranting confirmatory tests such as FibroScan or liver biopsy. The single-center study and small sample size limit the generalizability of our findings. Another limitation is using HSI to identify patients with hepatic steatosis in the clinic instead of ultrasonography. Nevertheless, our results support incorporating automated FIB-4 calculations into routine screenings. Further research that includes confirmatory diagnostic tests is needed.

## 5. Conclusions

Integrating the automated FIB-4 index into standard diabetes assessments may be useful for the case-finding of advanced liver fibrosis in patients with T2DM during routine medical care and enhance awareness of NAFLD among healthcare providers specializing in diabetes care. This approach can significantly improve clinical management and, ultimately, lead to better patient outcomes. Future studies should focus on optimizing risk prediction using these markers, determining their actual impact on clinical care and liver-related outcomes, and formulating strong evidence-based recommendations.

## Figures and Tables

**Table 1:** Clinical characteristics of patients with T2D and NAFLD seen at the diabetes clinic.

Characteristics	Total *n* = 318	Advanced Fibrosis *n* = 31	No Advanced Fibrosis *n* = 287	*p* Value
Age, years	54.8 ± 13.3	62.5 ± 10.9	53.9 ± 13.3	0.001
Sex (*n*, %)				
Male	144 (45.3)	15 (10.4)	129 (89.6)	
Female	174 (54.7)	16 (9.2)	158 (90.8)	
Saudi (*n*, %)	285 (89.6)	28 (9.8)	257 (90.2)	
HSI (*n* = 313)	42.2 ± 8.6	40.2 ± 9.6	42.4 ± 8.5	
No steatosis (<36) (*n*, %)	50 (16.0)	6 (12.0)	44 (88.0)	
Steatosis (>36) (*n*, %)	263 (84.0)	24 (9.1)	239 (90.9)	
BMI (kg/m^2^)	32.0 ± 6.7	30.1 ± 4.9	32.2 ± 6.8	
Hypertension (*n* = 316, %)				
Yes	177 (56.0)	20 (11.3)	157 (88.7)	
No	139 (44.0)	11 (7.9)	128 (92.1)	
Hemoglobin (g/dL)	12.9 ± 2.1	12.8 ± 2.3	12.9 ± 2.0	
Platelets (×10^9^/L)	263 (218–324)	167 (133–194)	272 (232–335)	<0.001
Bilirubin (mg/dL)	0.5 (0.3–0.7)	0.7 (0.5–1.0)	0.4 (0.3–0.6)	<0.001
Albumin (g/dL)	4.1 (3.8–4.3)	3.9 (3.6–4.1)	4.1 (3.8–4.3)	0.004
AST (IU/L)	17 (14–22)	25 (20–45)	17 (13–21)	<0.001
ALT (IU/L)	19 (13–28)	26 (16–53)	18 (13–27)	0.005
GGT (IU/L)	23 (16–34)	34 (17–144)	23 (16–33)	0.007
FBS (mg/dL)	131 (105–169)	139 (111–195)	130 (105–166)	
Hemoglobin A1c (*n*, %)	8.0 ± 1.9	7.6 ± 1.8	8.1 ± 1.9	
Total CHOL (mg/dL)	164.3 ± 43.5	147.8 ± 37.8	166.1 ± 43.8	0.026
LDL (mg/dL)	97 (72–129)	73 (66–118)	98 (75–129)	0.015
HDL (mg/dL)	43.8 ± 12.7	41.7 ± 13.2	44.0 ± 12.7	
TG (mg/dL)	114 (81–159)	107 (74–145)	115 (85–160)	
INR	1.00 (0.95–1.05)	1.06 (1.00–1.12)	0.99 (0.95–1.04)	<0.001
FIB-4	0.8 (0.6–1.2)	2.1 (1.6, 2.5)	0.7 (0.5–1.1)	<0.001
Medications (*n*, %)				
Insulin	172 (54.1)	20 (11.6)	152 (88.4)	
Metformin	244 (76.7)	21 (8.6)	223 (91.4)	
Sulfonylurea	17 (5.3)	1 (5.9)	16 (94.1)	
DPP-4 inhibitors (gliptins)	112 (35.2)	12 (10.7)	100 (89.3)	
SGLT2 inhibitors (gliflozins)	140 (44.0)	16 (11.4)	124 (88.6)	
GLP-1 agonist	97 (30.6)	9 (9.3)	88 (90.7)	
Pioglitazone	6 (1.9)	2 (33.3)	4 (66.7)	
Statin	254 (79.9)	28 (11.0)	226 (89.0)	
Ezetimibe	39 (12.3)	4 (10.3)	35 (89.7)	

The *p* value compares patients with and without advanced fibrosis. Data are expressed as the mean (±SD), median (interquartile range), or *n* (%). AF, advanced fibrosis; HSI, hepatic steatosis index; AST, aspartate aminotransferase; ALT, alanine transaminase; GGT, gamma-glutamyl transferase; IU, international units; L, liter; FBS, fasting blood sugar; CHOL, cholesterol; TG, triglycerides; HDL, high-density lipoprotein; LDL, low-density lipoprotein; INR, international normalized ratio; FIB-4, fibrosis-4 index; DDP-4, dipeptidyl peptidase-4 inhibitors; SGLT2, sodium-glucose co-transporter-2 inhibitors; GLP-1 agonist, glucagon-like peptide-1 agonist.

**Table 2:** Multivariate logistic regression analysis of variables associated with the development of advanced fibrosis in patients with type 2 diabetes.

Variable	OR	95% CI	*p* Value
Age (years)	1.06	1.02–1.10	0.003
Elevated total bilirubin (>1 mg)	4.86	1.55–15.19	0.007
Elevated GGT (>55 IU/mL)	5.16	2.10–12.68	<0.001
Prolonged INR (>1.2)	4.18	1.17–14.96	0.028

OR, odds ratio; CI, confidence interval; INR, international normalized ratio; GGT, gamma-glutamyl transferase.

## Data Availability

The data presented in this study are available upon request from the corresponding author.
